# Supplementary material for: Inflammatory factors and risk of lung adenocarcinoma: a Mendelian randomization study mediated by blood metabolites
Source: Front Endocrinol (Lausanne). 2024 Aug 27;15:1446863. doi: 10.3389/fendo.2024.1446863 (PMC11384989; doi:10.3389/fendo.2024.1446863)
Supplement: Supplementary file 1 [file DataSheet1.zip › supplementary files/S2/GCST90200205/scatter.pdf]

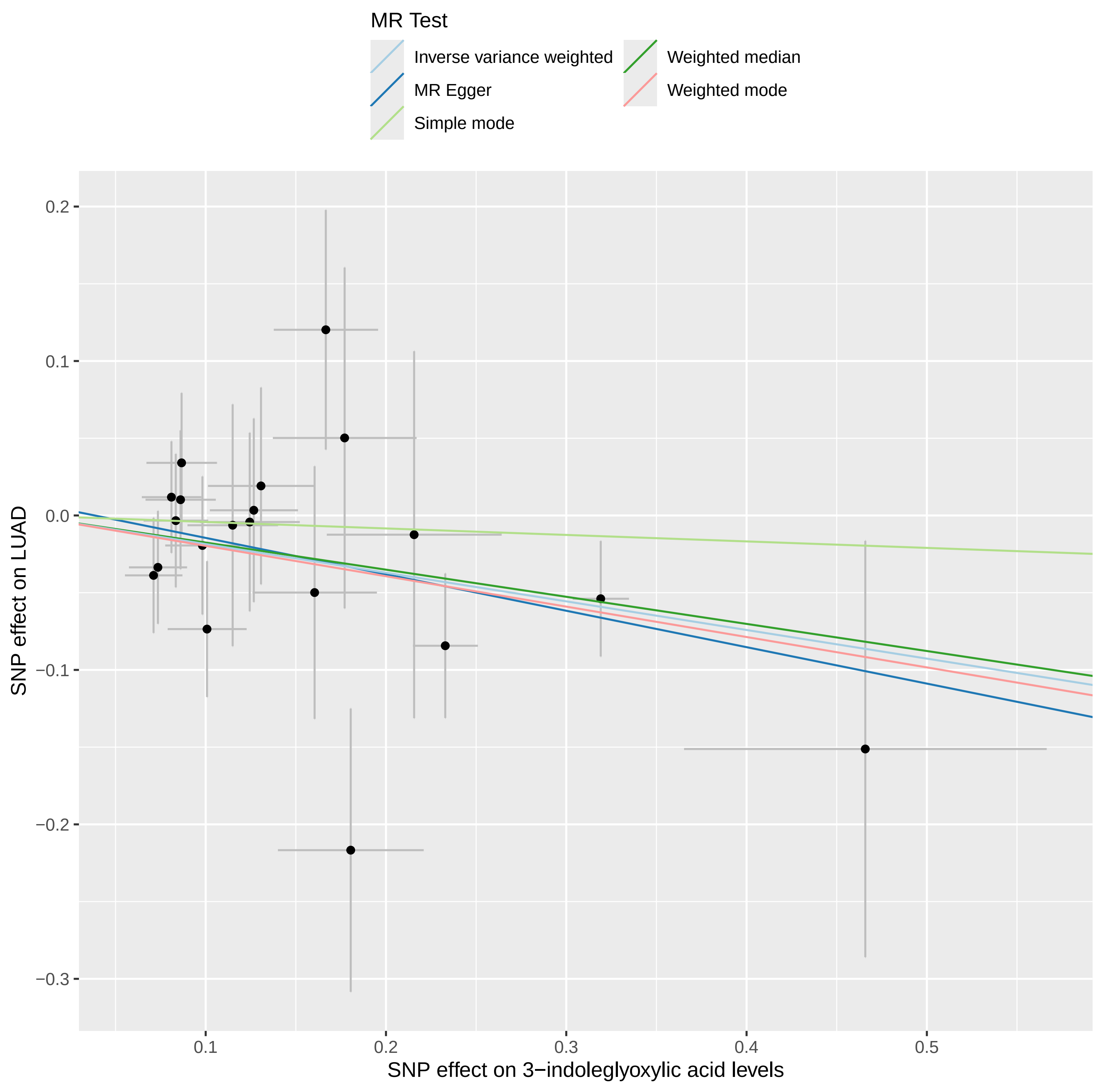

MR Test
Inverse variance weighted
MR Egger
Simple mode
Weighted median
Weighted mode
0.2
0.1
0.0
−0.1
−0.2
−0.3
SNP effect on LUAD
0.1
0.2
0.3
0.4
0.5
SNP effect on 3−indoleglyoxylic acid levels
